# When Organic Chemistry Meets Surface Science: From Nanographene Characterization to Single-Molecule Reactions

**DOI:** 10.1021/jacs.6c00918

**Published:** 2026-10-22

**Authors:** Diego Peña, Iago Pozo, Dolores Pérez

**Affiliations:** † Centro de Investigación en Química Biolóxica e Materiais Moleculares (CiQUS) and Departamento de Química Orgánica, 16780 Universidade de Santiago de Compostela, Santiago de Compostela 15782, Spain; ‡ Oportunius, Galician Innovation Agency, Santiago de Compostela 15782, Spain

## Abstract

The interplay between organic chemistry and surface science allows for atomically precise characterization, synthesis, and manipulation of carbon-based nanostructures. Advances in scanning probe microscopy and on-surface synthesis have unlocked new routes to obtaining fascinating nanographenes, exotic and highly reactive molecules, and controlled single-molecule reactions. Importantly, these developments open access to transformations that are inaccessible to solution-phase chemistry. Here, we illustrate the potential of this approach and outline the future opportunities and challenges.

## Graphical abstract

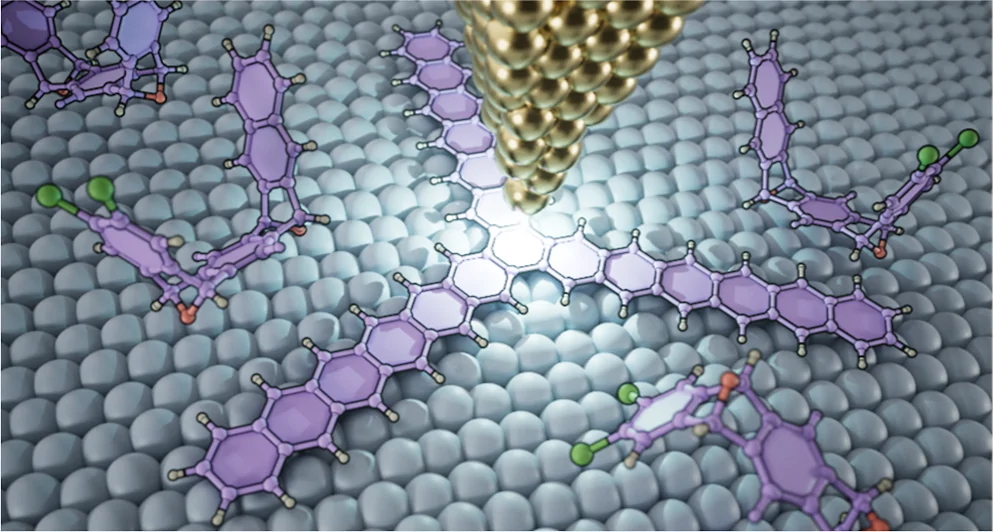

## Introduction

In 1959, in his famous lecture “Plenty of Room at the Bottom”, Richard Feynman envisioned a future in which the atom-by-atom construction of single molecules would become possible: “···it would be, in principle, possible (I think) for a physicist to synthesize any chemical substance that the chemist writes down. Give the orders and the physicist synthesizes it. How? Put the atoms down where the chemist says, and so you make the substance. The problems of chemistry and biology can be greatly helped if our ability to see what we are doing, and to do things on an atomic level, is ultimately developed—a development which I think cannot be avoided.”

Now, 67 years later, this prediction has become a reality through the convergence of two classical scientific disciplines: organic chemistry and surface science.

This perspective focuses on the evolution and recent advances at the interface of these two disciplines that have enabled the resolution of long-standing chemical challenges (Figure 1a). To take advantage of this interdisciplinarity, advances in scanning probe microscopy (SPM) were essential. In this respect, submolecular resolution in SPM of on-surface-deposited molecules has proven crucial and was made possible by the introduction of functionalized tips in atomic force microscopy (AFM) operated under ultrahigh vacuum (UHV) and at cryogenic temperatures (Figure 1b,c).^1^ In fact, AFM has evolved into a powerful tool for the identification of individual molecules.^2^ Beyond structural assignment, it can reveal key molecular features such as conformation, charge state, and charge distribution and, when combined with scanning tunneling microscopy (STM), enable the analysis of frontier orbital occupancy and the visualization of their electron density. A pioneering work by Gross and co-workers at IBM Research Zurich has established AFM as an advanced method for molecular structure elucidation.^3^ For example, in 2012, it was demonstrated that AFM with CO-terminated tips can discriminate bond orders in molecules adsorbed on bilayer NaCl on Cu(111) with high precision, based on two complementary effects: the bond-dependent frequency shift and the apparent bond length observed in constant height AFM images.^4^ The former is directly related to variations in electron density, which increase with bond order; higher electron densities give rise to stronger Pauli repulsion, resulting in larger frequency shifts. The latter effect arises from the tilting of the CO-terminated tip, which amplifies the apparent bond-length differences. On insulating multilayer NaCl films, high-resolution AFM images of organic molecules in neutral, cationic, and anionic states were obtained, allowing changes in conformation, adsorption geometry, and bond-order relationships to be detected as a function of the molecular charge state.^5^

**Figure 1:**
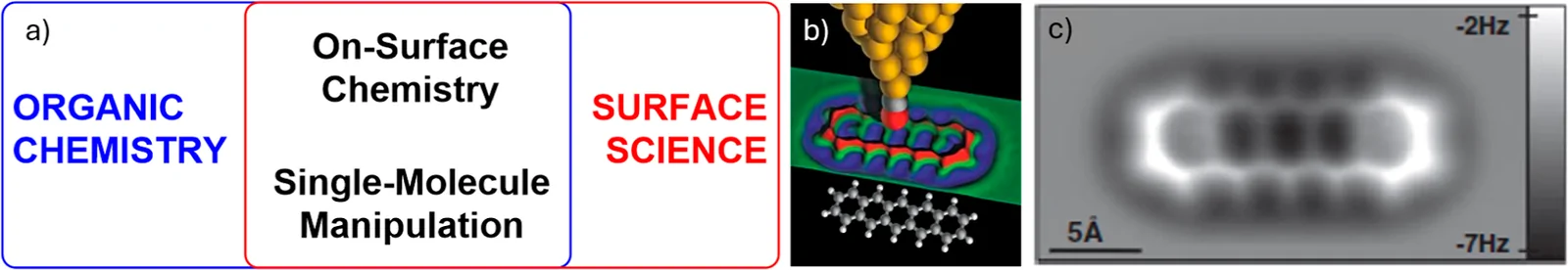
(a) Schematic representation of the interface between organic chemistry and surface science. (b) Illustration of the interaction between a single pentacene molecule on a surface and a CO-functionalized AFM tip.^2^ Reprinted with permission from ref 2. Copyright 2011 Springer Nature. (c) AFM image of an individual pentacene molecule on Cu(111) at 5 K.^1^ Reprinted with permission from ref 1. Copyright 2009 AAAS.

In parallel, computational methods, particularly density functional theory calculations, have played a central role in the interpretation of STM/AFM experiments by providing adsorption geometries, electronic structures, simulated images, and reaction pathways for surface-confined transformations. The close interplay between experiments and theories has therefore become essential for understanding reactivity and molecular properties on surfaces.

### Nanographene Characterization

The methodological advances mentioned in the introduction paved the way for the application of bond-resolved AFM to the characterization of extended π-conjugated systems, most notably molecular nanographenes, where direct real-space imaging provides access to bond connectivity with single-molecule precision. Nanographenes are finite graphene-like molecules composed of fused aromatic rings, whose electronic, optical, and magnetic properties are strongly governed by their size, geometry, and edge structure.^6^ Bond-resolved AFM has proven essential for the unambiguous characterization of unsubstituted large polycyclic aromatic hydrocarbons (PAHs) and nanographenes synthesized by solution chemistry, for which conventional spectroscopic techniques often fail due to the limited solubility of these molecules. For example, the 3-fold-symmetric nanographene **1**, formed by the fusion of 22 benzene rings, was obtained by Pd-catalyzed aryne cyclotrimerization.^7^ The extreme insolubility of this compound precluded structural confirmation by NMR spectroscopy. However, AFM analysis with a CO-functionalized tip after deposition on bilayer NaCl on Cu(111) enabled a definitive structural assignment (Figure 2a). Interestingly, AFM inspection of additional adsorbates revealed unexpected compounds. This finding highlighted the power of AFM for reaction-mixture analysis, as on-surface identification of byproducts yielded critical insights into solution-phase reaction mechanisms.

**Figure 2:**
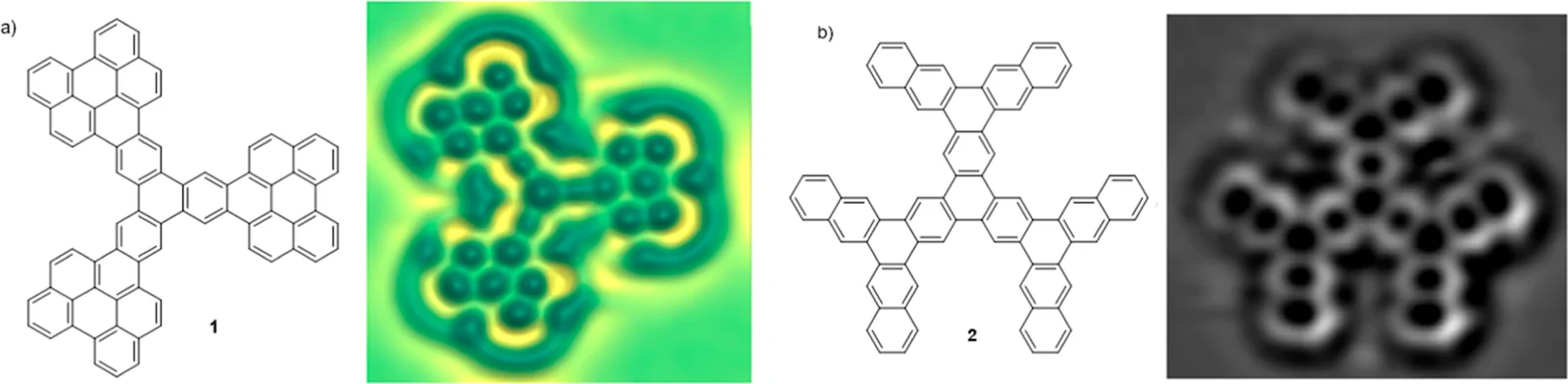
(a) Structure of the 3-fold-symmetric nanographene **1** and AFM image.^7^ Reprinted with permission from ref 7. Copyright 2014 Wiley VCH. (b) Structure of [19]­dendriphene **2** and the corresponding AFM image.^8^ Reprinted with permission from ref 8. Copyright 2018 Wiley VCH.

Similarly, the 19-ring dendritic nanographene **2** ([19]­dendriphene) was also obtained by Pd-catalyzed aryne cyclotrimerization and characterized by AFM (Figure 2b).^8^ The electronic characterization of individual dendriphene **2** molecules was further investigated by STM, revealing the spatial distribution of the highest occupied molecular orbital and the lowest unoccupied molecular orbital. Scanning tunneling spectroscopy (STS) enabled determination of the HOMO–LUMO transport gap of molecules on surfaces. As expected, based on the presence of nine Clar sextets in nanographene **2**, STS showed a substantial transport gap of 4.1 eV, considerably larger than those reported for other families of *cata*-fused PAHs such as acenes.

High-resolution SPM enables the molecular characterization of individual constituents within complex mixtures. In 2015, atomic-resolution AFM was applied to study asphaltenes, the solid fraction of crude oil composed of highly diverse polyaromatic molecules.^9^ Long-standing limitations of conventional techniques had left their molecular architecture unresolved; AFM revealed extraordinary structural diversity, while complementary STM orbital imaging enabled a detailed structural assignment. Similarly, atomic-resolution AFM has been employed for the identification of fuel pyrolysis products,^10^ the visualization of individual PAHs in meteorites,^11^ and the detection of intermediates in soot nanoparticle formation.^12^

### On-Surface Cyclodehydrogenation

While high-resolution AFM has demonstrated remarkable capabilities for the structural characterization of complex polyaromatic systems, the structural diversity accessible for nanographenes synthesized by conventional solution chemistry remains inherently limited. To overcome these constraints, on-surface synthesis has emerged as a powerful strategy for increasing the structural complexity of nanographenes, enabling the bottom-up fabrication of atomically precise graphene-based nanostructures with tailored size, shape, and edge topology.^13–17^ In on-surface synthesis, tailor-made molecular precursors are deposited onto a well-defined surface (frequently a metallic one), where surface-assisted and thermally activated reactions proceed under spatial confinement, allowing the formation of atomically precise molecular nanostructures that are often inaccessible by solution chemistry.

In this respect, one of the first reactions introduced in the field of on-surface synthesis was cyclodehydrogenation, also referred as intramolecular oxidative aromatic coupling, in which multiple C_aryl_–C_aryl_ bonds are formed in a single operation to yield a large PAHs or nanographenes.^18,19^ While in solution chemistry, this transformation is typically promoted by strong oxidants and requires harsh reaction conditions (Scholl reaction), its on-surface counterpart can be achieved simply by thermal annealing under UHV conditions of a suitably designed polyphenylene precursor adsorbed on Au(111). Figure 3 illustrates two representative examples highlighting the potential of this on-surface transformation to obtain nonplanar porous nanographenes **4** ^20^ and **6**,^21^ starting from substituted starphene precursors **3** and **5**, respectively.

**Figure 3:**
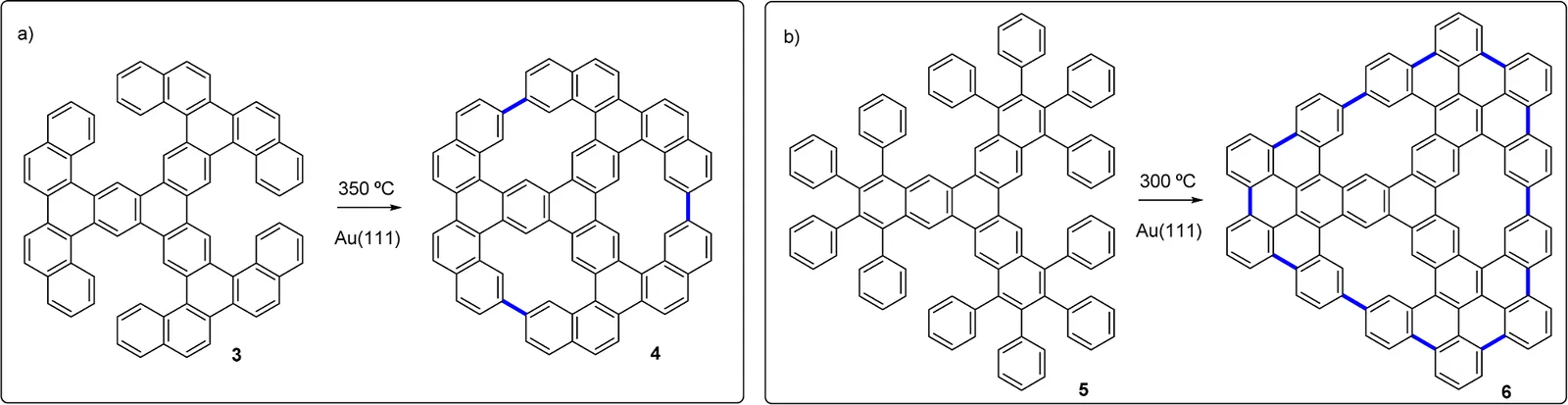
(a) Synthesis of nonplanar porous nanographene **4** by on-surface cyclodehydrogenation.^20^ (b) Synthesis of nonplanar porous nanographene **6**.^21^ Bonds formed during the cyclodehydrogenation reaction are highlighted in blue.

Other examples in which surface-assisted cyclodehydrogenation serves as a key reaction step include the preparation of peripentacene,^22^ periheptacene,^23^ and dibenzoperihexacene,^24^ among other nanographenes.^25^

More broadly, surface-assisted cyclodehydrogenation is not restricted to coupling between sp^2^-hybridized carbon atoms. Intramolecular cyclodehydrogenative coupling involving sp^3^-hybridized carbon atoms has also been demonstrated upon thermal treatment of dodecamethylhexabenzocoronene on a Cu(111) surface, leading to the formation of circumcoronene.^26^ In this case, additional dehydrogenation steps following the cyclization reactions are required to achieve the fully aromatized final product. In addition, thermally induced cyclodehydrogenative sp^2^–sp^3^ coupling followed by successive dehydrogenation steps has been demonstrated as a versatile route to obtain a broad diversity of nanographenes. This approach has been extensively employed to access open-shell nanographenes, such as triangulenes, synthesized on surfaces.^27^ Under UHV conditions, these nanographenes with pronounced radical character are remarkably stable, providing a unique opportunity to investigate their magnetic properties, including inelastic spin excitations. For example, macrocycle **8**, composed of six [3]­triangulene moieties, was obtained by thermal annealing of compound **7** deposited on Au(111) (Figure 4a). The resulting triangulene nanostar **8** exhibits a collective spin state arising from the interaction of its 12 unpaired π-electrons, corresponding to antiferromagnetic coupling between six *S* = 1 units, one associated with each triangulene subunit.^28^ More recently, on-surface annealing of compound **9** deposited on Au(111) led to the formation of the extended aza-triangulene **10** through the formation of 12 new C–C bonds (Figure 4b). Supported by theoretical calculations, the three radical units were found to be localized at distinct regions of the molecule and to couple via symmetric ferromagnetic interactions, resulting in a *S* = 3/2 ground state.^29^

**Figure 4:**
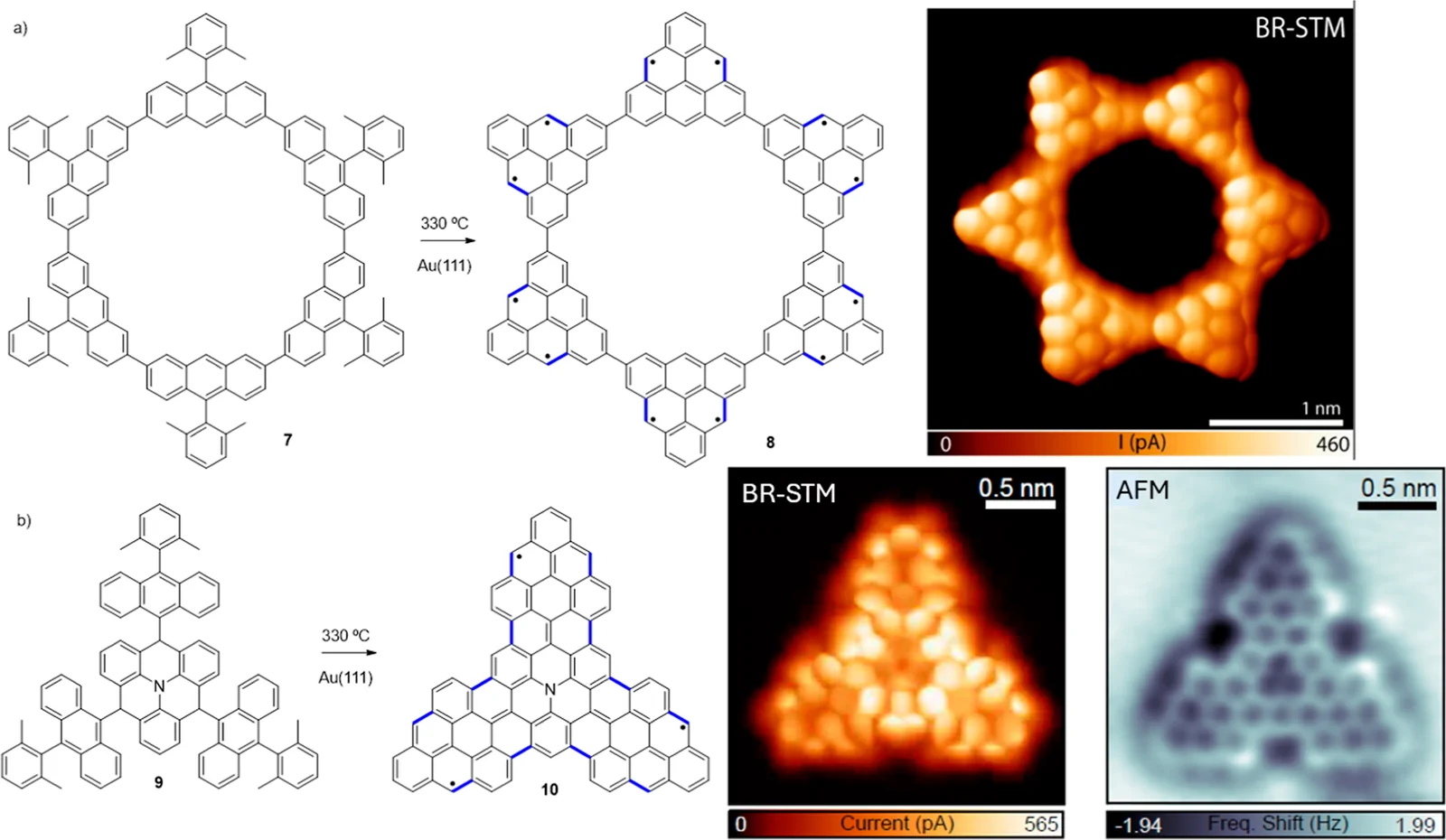
(a) On-surface synthesis of a macrocycle formed by six [3]­triangulenes and bond-resolved STM image (BR-STM).^28^ Reprinted with permission from ref 28. Copyright 2021 Wiley VCH. (b) On-surface synthesis of an extended aza-triangulene and BR-STM/AFM images.^29^ Bonds formed during the cyclodehydrogenation reaction are highlighted in blue. Reproduced from ref 29. Available under a CC-BY 4.0 license. Copyright 2025 ACS.

Additional examples of the on-surface preparation of open-shell nanographenes via intramolecular cyclodehydrogenative coupling include the synthesis of [4]­triangulene,^30^ [5]­triangulene,^31^ [7]­triangulene,^32^ Clar’s goblets,^33,34^ and rhombenes,^35^ as well as numerous other triangulene derivatives.^36–40^ This reaction strategy has also been applied to the on-surface synthesis of π-extended phenalenyl-porphyrins exhibiting open-shell character^41^ and has been further extended to the formation of C–N bonds.^42^

While metallic substrates play a key catalytic role in cyclodehydrogenative planarization, they limit applications that rely on the intrinsic electronic properties of nanographenes. To address this challenge, alternative strategies have been developed to induce cyclodehydrogenation on nonmetallic surfaces, including atomic hydrogen-assisted cyclodehydrogenations on semiconducting or insulating substrates^43^ and HF-zipping reactions enabled by fluorinated precursors on TiO_2_.^44^

### On-Surface Ullmann-like Coupling

Whereas cyclodehydrogenation dominates intramolecular C–C bond formation in on-surface synthesis, Ullmann-like coupling represents the most frequently employed strategy for intermolecular C–C coupling. Originally introduced at the beginning of the twentieth century as a copper-mediated solution-phase reaction for the homocoupling of aryl halides,^45^ the Ullmann-like reaction was later adapted to surfaces, where dehalogenation of adsorbed aryl halides on metal substrates enables intermolecular C–C bond formation under UHV conditions. This surface-assisted transformation, first clearly demonstrated in 2007 by Grill, Hecht, and co-workers,^46^ established a general strategy for the bottom-up fabrication of covalent nanostructures and became a fundamental reaction in on-surface synthesis.^47,48^

Since this seminal work, surface-assisted Ullmann-like coupling has been widely exploited to construct one- and two-dimensional covalent nanostructures with atomic precision. In particular, the combination of Ullmann-like coupling as a privileged on-surface polymerization step with cyclodehydrogenation for subsequent planarization of the resulting polymers has led to a vast family of graphene-based derivatives. In 2010, Fasel, Müllen, and co-workers demonstrated this approach to obtain atomically precise graphene nanoribbons.^49^ Since then, this strategy has become the dominant bottom-up route for the fabrication of graphene nanostructures with well-defined width, edge structure, and electronic properties.^13,50–55^

Careful precursor design enables the preparation of fascinating nanostructures with advanced properties through the combination of Ullmann-like coupling and cyclodehydrogenation. For example, thermal treatment of dibrominated triphenylene **11** deposited on Au(111) at 200 °C leads to the formation of macrocycle **12**, which upon further annealing at 350 °C undergoes cyclodehydrogenation to yield the porous nanographene **13** (Figure 5a).^56^ Analogous thermal treatment of compound **14**, featuring two bromine substituents oriented at 120°, resulted in the formation of graphene nanoring **15** via the fusion of six precursor molecules (Figure 5b).^57^ Related compounds were prepared following a similar strategy.^58–60^

**Figure 5:**
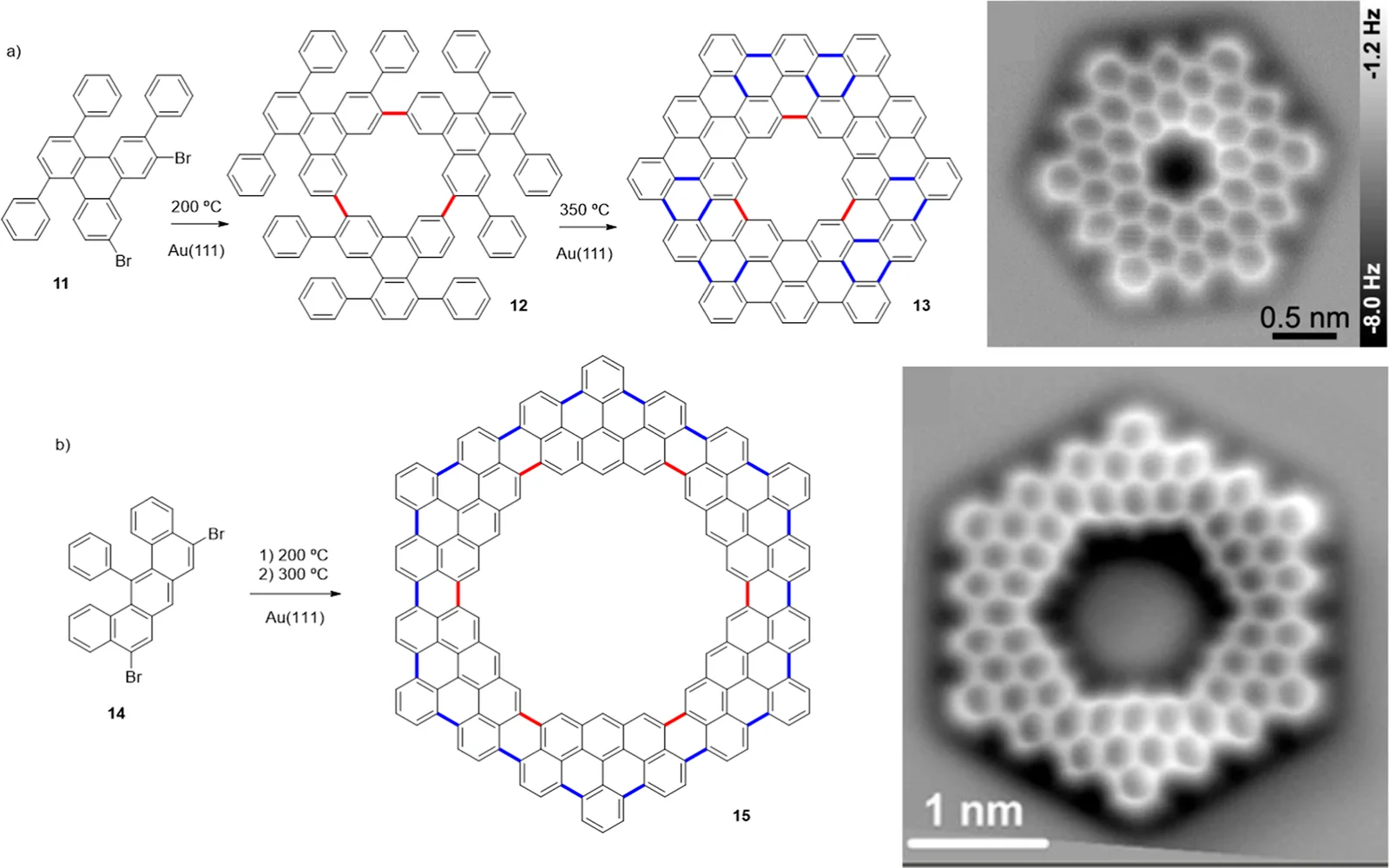
(a) Formation of porous nanographenes by combining surface-assisted Ullmann-like couplings and cyclodehydrogenations. (a) Sequential annealing of dibrominated triphenylene **11** on Au(111) to yield double-stranded hexagonal cycloarene **13**.^56^ Reprinted with permission from ref 56. Copyright 2020 American Chemical Society. (b) Analogous on-surface coupling of precursor **14** to form hexagonal coronoid **15**.^57^ Bonds formed by Ullmann-like coupling and cyclodehydrogenation are highlighted in red and blue, respectively. Reproduced from ref 57. Available under a CC-BY 4.0 license. Copyright 2020 ACS.

### Alternative Surface-Assisted Reactions

Beyond Ullmann-like reactions and cyclodehydrogenations, a variety of additional on-surface transformations have been developed to expand the synthetic toolbox of on-surface chemistry.^16^ Representative examples include the cyclization of enediynes,^61^ the homocoupling of terminal alkynes,^62^ and carbene coupling promoted by =CBr_2_ functionalities.^63^

Deoxygenation represents another reaction that has been demonstrated on metallic surfaces. Using this approach, thermal treatment of pentaepoxydodecacene **16** (Figure 6a) on Au(111) affords dodecacene (**17**).^64^ Large acenes are linear *cata*-condensed PAHs with unique electronic properties. Although they are highly unstable under ambient conditions—consistent with a structure containing only one Clar sextet—it is nevertheless possible to generate and electronically characterize them under UHV conditions. Additional strategies for the on-surface generation of large acenes include dehydrogenation reactions, such as the conversion of compound **18** into tridecacene (**19**),^65^ as well as acetylene extrusion processes involved in the transformation of compound **20** into pentadecacene (**21**), which represents the largest unsubstituted acene obtained to date.^66^ Although the general trend in acene series is a reduction of the HOMO–LUMO gap with increasing molecular length, STS measurements reveal that the gap saturates and does not decrease below approximately 1.1 eV.^66^ By contrast, a starphene geometry can lead to PAHs with reduced transport gaps. For example, [19]­starphene (**23**), obtained by deoxygenation of compound **22** (Figure 6b), displays an STS gap of 0.9 eV.^67^ An important remaining challenge in on-surface synthesis is the expansion of the available reaction repertoire, which would enable the preparation of even more complex structures with advanced properties. The most common strategy has been to adapt well-established reactions from solution-phase organic chemistry to surface environments. However, this approach does not always succeed, as the mechanistic requirements on surfaces differ substantially from those in solution. The Diels–Alder reaction exemplifies this limitation, as only transannular reactions of strained precursors proceed efficiently on surfaces.^68^ By contrast, other transformations, such as cyclotetramerization of phthalonitriles to obtain phthalocyanines,^69,70^ are fully compatible with surface confinement.

**Figure 6:**
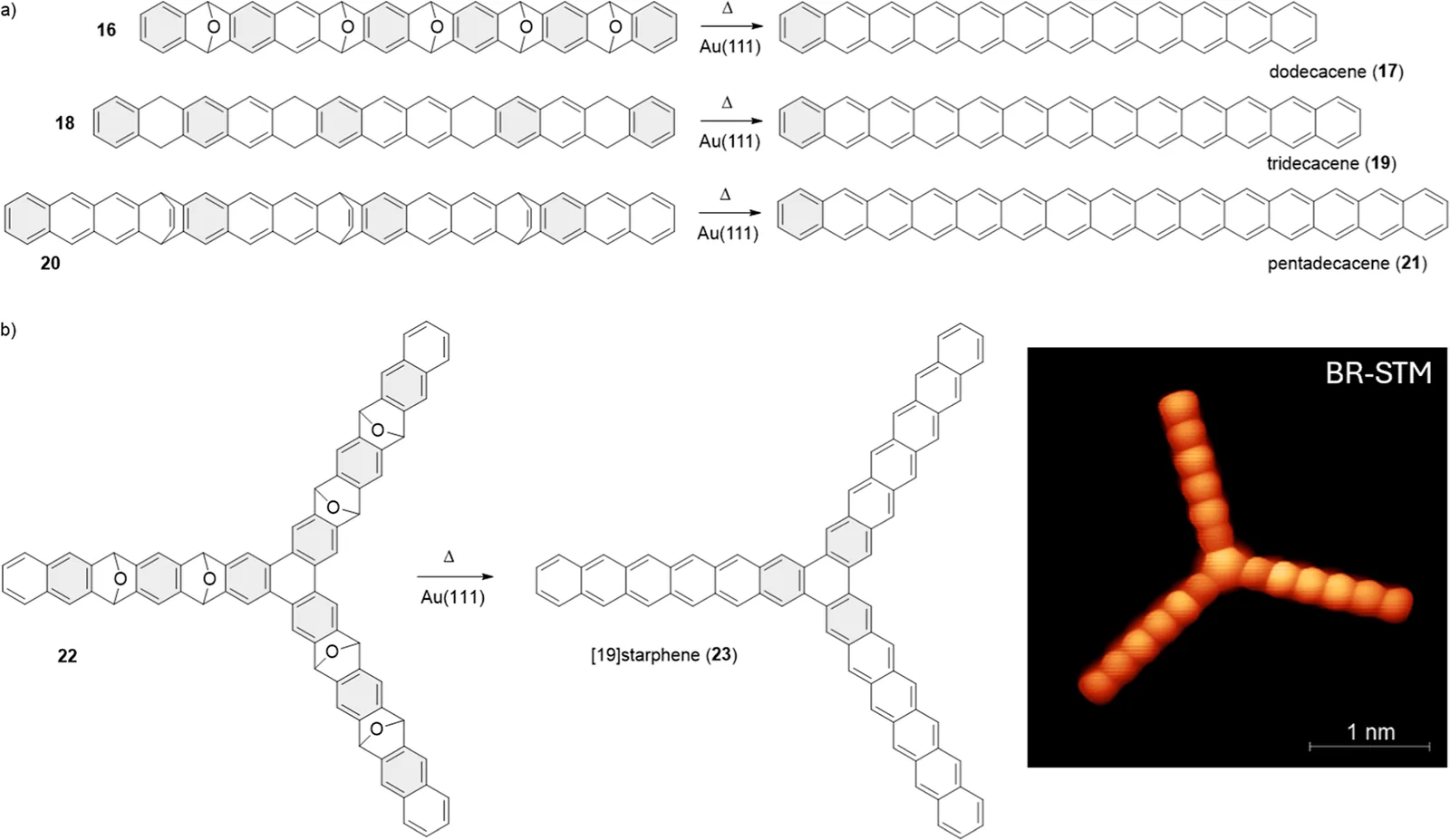
(a) On-surface generation of large acenes. (b) On-surface generation of [19]­starphene and BR-STM image.^67^ Reprinted with permission from ref 67. Copyright 2024 Wiley VCH. Clar sextets are highlighted in gray.

Even more interesting is the development of entirely new on-surface reactions that have no precedent in solution-phase chemistry. Figure 7 highlights three recent and representative examples. One such example is the defluorinative coupling of CF_3_-substituted porphyrins, such as **24**, which leads to the formation of covalent polymers **25** (Figure 7a).^71^ Another example is the dimerization of isopropyl moieties on a gold surface, as illustrated by the transformation of compound **26** into polymer **27** (Figure 7b).^72^ This process corresponds to a formal (3 + 3) cycloaromatization, resulting in the formation of a new benzene ring. Finally, indenyl coupling has been demonstrated in the conversion of compound **28** into the polyacetylene derivative **29** through a regio- and stereoselective polymerization process (Figure 7c).^73,74^

**Figure 7:**
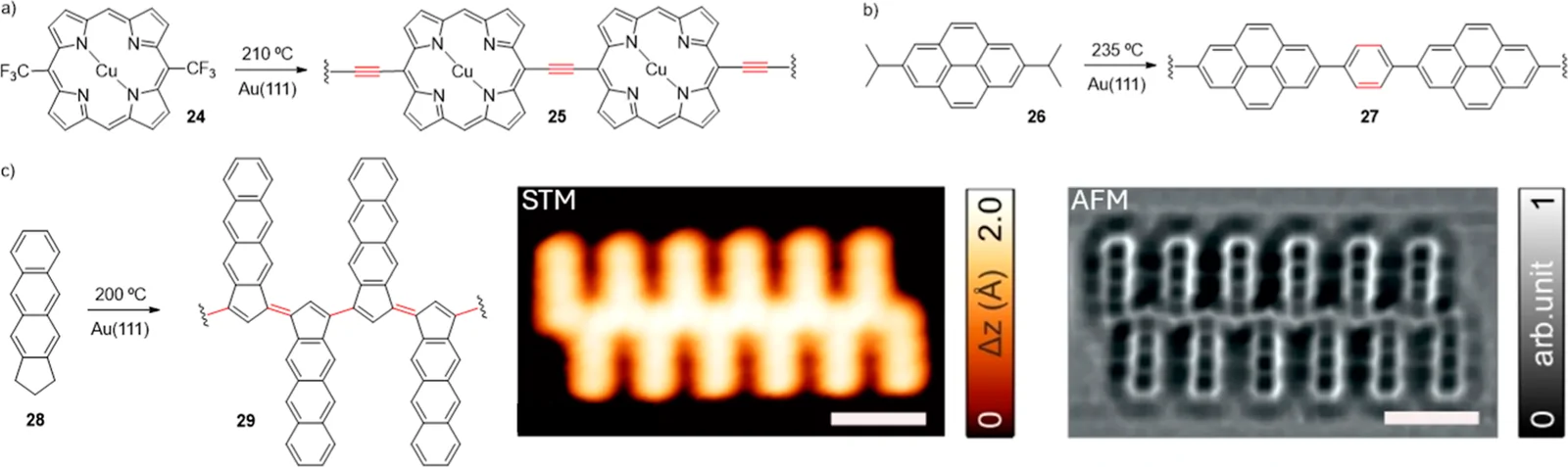
(a) Defluorinative coupling of CF_3_-substituted porphyrins.^71^ (b) (3 + 3) Cycloaromatization of isopropyl moieties.^72^ (c) Regio- and stereoselective polymerization through indenyl coupling. STM and AFM images of polymer **29**.^73^ Reprinted with permission from ref 73. Copyright 2025 Springer Nature.

Together, these examples underscore how surface confinement enables entirely new reaction pathways that greatly expand the synthetic scope of on-surface chemistry beyond the limits of solution phase reactions. Although the initial development of this field primarily focused on nanographene synthesis, these methodologies have since expanded far beyond such systems to the preparation of diverse advanced molecular materials.

### Single Molecule Reactions

Most of the transformations discussed above are thermally induced reactions carried out on metallic surfaces. An alternative approach that more closely reflects the original vision articulated by Feynman (see Introduction) is to induce chemical transformations by atomic manipulation, that is, by triggering reactions using the tip of STM/AFM.^3^ As early as 2000, an Ullmann-like coupling reaction was demonstrated at the single-molecule level to form biphenyl from iodobenzene on Cu.^75^ In this pioneering experiment, voltage pulses were first used to induce C–I bond dissociation, generating phenyl radicals. Two such radicals were then manipulated and brought together to form a biphenyl molecule. More recently, tip-induced Ullmann-like coupling has been employed to construct a covalent organic nanoarchitecture molecule by molecule on insulating NaCl surfaces.^76^ In addition, C–H cleavage reactions triggered by voltage pulses from an STM tip have been demonstrated, for example, to generate [3]­triangulene.^27^ Notably, this concept has been extended to the automated synthesis of larger triangulenes, where artificial intelligence was used to guide and execute tip-induced reaction sequences.^77^

Beyond Ullmann-like coupling and cyclodehydrogenation, several additional transformations have been demonstrated through the atomic manipulation. For example, tip-induced dehalogenation has been used to generate short-lived reactive species such as *o*-arynes on NaCl surfaces, which were subsequently characterized by AFM.^78^ When this strategy was applied to dibromoanthracene (**30**), the *p*-aryne **32** is formed by two successive tip-induced debromination reactions (Figure 8a).^79^ By applying voltage pulses from the STM tip, this *p*-diradical can be reversibly interconverted into the 10-membered ring diyne **33** and back, thus constituting a reversible retro-Bergman cyclization driven by tip-induced voltage pulses.

**Figure 8:**
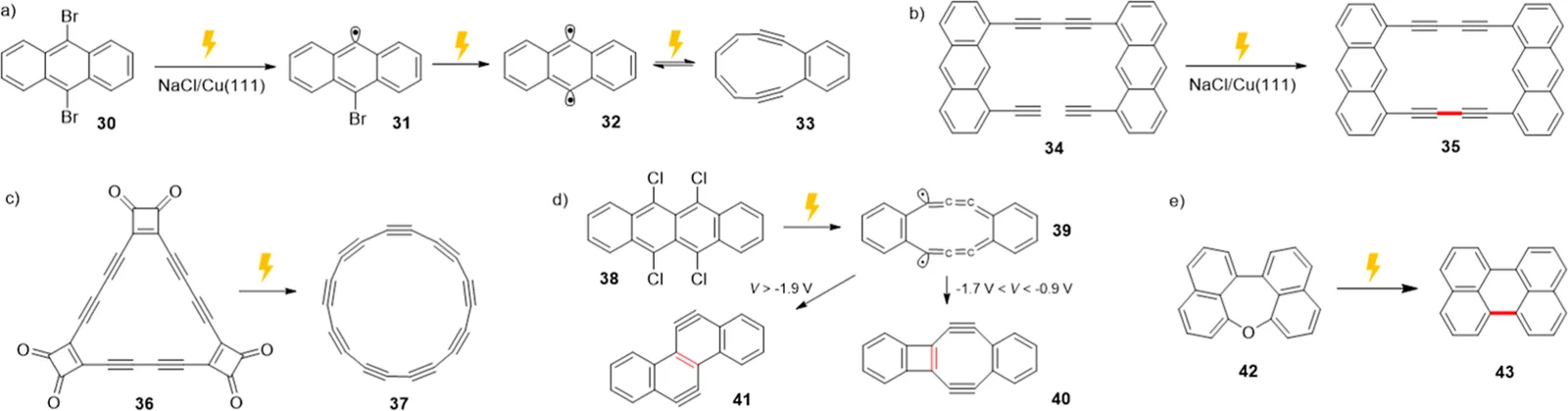
Selected examples of tip-induced chemistry on single molecules. (a) *para*-Aryne generation and reversible retro-Bergman cyclization.^79^ (b) Intramolecular Glaser-type coupling.^80^ (c) Generation of cyclo[18]­carbon.^81^ (d) Rearrangement cascade and selectivity in a single-molecule reaction.^85^ (e) Oxygen deletion by tip-induced chemistry.^87^

Tip-induced chemistry has enabled not only bond dissociation reactions but also the formation of new covalent bonds in single molecules. For example, compound **34** was converted into **35** through the formation of a new C–C bond between terminal alkynes in a Glaser-type coupling reaction (Figure 8b).^80^

A particularly notable application of this strategy is the generation of carbon rings composed exclusively of sp-hybridized carbon atoms. In 2019, this was first demonstrated through the generation of cyclo[18]­carbon (**37**) by a sequence of tip-induced voltage pulses applied to molecular precursor **36** deposited on a NaCl surface (Figure 8c).^81^ Following this pioneering work, the family of cyclocarbons accessible by on-surface synthesis has expanded rapidly in recent years.^82–84^

Rearrangement cascades at the single-molecule level have also been demonstrated by tip-induced chemistry. Notably, it was shown that reaction selectivity can be achieved within an individual molecule by tuning the amplitude and the sign of the applied voltage pulse. In particular, tetrachlorotetracene **38** is first converted into intermediate **39**, which can be selectively transformed into either compound **40** or **41** depending on the pulse amplitude (Figure 8d).^85^

Tip-induced chemistry can be regarded as an ideal strategy for performing skeletal editing in a straightforward and highly controlled manner.^86^ This concept was recently demonstrated by the transformation of oxepine **42** into perylene (**43**) on NaCl by means of voltage pulses, a process that can be described as a tip-induced oxygen deletion reaction (Figure 8e).^87^

Together, these advances demonstrate that tip-induced chemistry enables unprecedented control over bond cleavage, bond formation, and skeletal editing at the single-molecule level, bringing Feynman’s vision of atom-by-atom synthesis closer to reality.

## Conclusions

In summary, the convergence of organic chemistry and surface science has enabled unprecedented insights into the structure and reactivity of carbon-based nanostructures. The development of on-surface synthesis, together with advances in SPM, has made it possible to both construct and directly characterize complex nanographenes and reactive intermediates with an atomic precision. While classical transformations such as cyclodehydrogenation and Ullmann-like coupling remain central to the field, a growing number of surface-specific and tip-induced reactions have considerably broadened the accessible chemical space. These approaches allow the controlled generation of open-shell systems, carbon allotropes, and single-molecule transformations that cannot be achieved under conventional solution phase conditions. Overall, on-surface synthesis has evolved into a complementary chemical toolbox that not only extends established synthetic concepts but also provides access to new forms of reactivity and molecular architectures.

Despite the significant advances achieved in on-surface synthesis, several important challenges remain. A major limitation is the strong dependence on metallic substrates, which often facilitate reactivity and visualization, but can restrict access to the intrinsic properties of the resulting nanostructures. In fact, metallic surfaces, such as Au(111) and Cu(111), efficiently promote C-halogen bond activation, giving rise to a broad range of accessible reactions. However, their strong interaction with the resulting nanostructures can significantly perturb the intrinsic electronic and magnetic properties of this system. By contrast, although the lower reactivity of nonmetallic surfaces represents a significant limitation, the synthesis of functional nanostructures on semiconducting and insulating substrates could facilitate the development of molecular-based technologies, by providing improved control over the electronic and magnetic states, which could in principle be addressed spectroscopically. Strategies such as the addition of catalytic amounts of metals or dosing with atomic hydrogen could facilitate the development of on-surface synthesis on nonmetallic substrates.^43^ Another attractive strategy to avoid the use of metallic surfaces is the implementation of photochemical methods in place of thermal activation to induce on-surface transformations. Although only a few pioneering examples have been reported so far,^88–90^ the use of light as an external stimulus is expected to significantly expand the available synthetic toolbox.

Further challenges arise from the limited scalability of on-surface methods and from the fact that many of the most precise strategies operate at the single-molecule level. Translating these capabilities into methods that can produce macroscopic quantities of material or operate under more practical environmental conditions will require further instrumental and technological developments.

Beyond these aspects, the automation of SPM characterization and manipulation under UHV conditions is still in its infancy,^91–95^ while extending these concepts to three-dimensional molecular architectures or biologically relevant systems introduces additional complications related to deposition, adsorption, and conformational freedom.^96,97^ Addressing these issues will be essential for fully exploiting the interplay between organic chemistry and surface science to tackle increasingly complex and impactful scientific challenges.
